# Supplementary figures and images for: Impact of non-pharmaceutical interventions targeted at the COVID-19 pandemic on the incidence of influenza-like illness in the UK Armed Forces
Source: PLoS One. 2022 Dec 1;17(12):e0270438. doi: 10.1371/journal.pone.0270438 (PMC9714820; doi:10.1371/journal.pone.0270438)

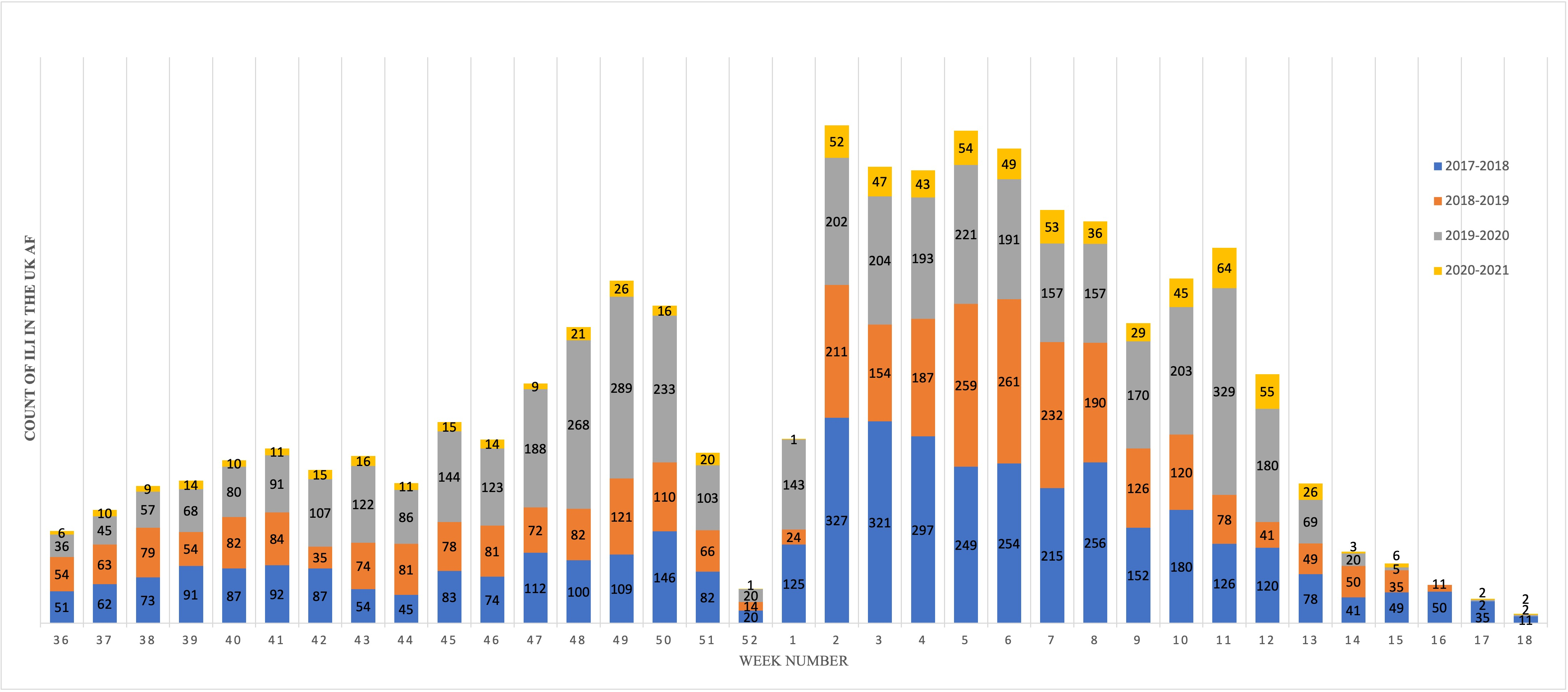

Supplement: S1 Fig — (TIFF) [file pone.0270438.s001.tiff]
